# Long-Term Use of Proton-Pump Inhibitors: Unravelling the Safety Puzzle

**DOI:** 10.7759/cureus.52773

**Published:** 2024-01-23

**Authors:** Manish S Bhatnagar, Sachin Choudhari, Dattatray Pawar, Akhilesh Sharma

**Affiliations:** 1 Gastroenterology, Icon Hospital, Ahmedabad, IND; 2 Medical Affairs, Alkem Laboratories Ltd., Mumbai, IND

**Keywords:** cardiovascular, pantoprazole, risks, long-term use, safety concerns, proton-pump inhibitors

## Abstract

Globally, over 25% of the population suffers from acid-related disorders such as dyspepsia or gastroesophageal reflux disease (GERD), and around 7.6% of Indians report having GERD symptoms on a frequent enough basis to warrant a diagnosis. Over the past three decades, proton-pump inhibitors (PPIs) have been the mainstay of medical therapy for acid-peptic diseases like GERD, etc. Additionally, they are frequently prescribed for prophylactic purposes and in conjunction with non-steroidal anti-inflammatory drugs. PPIs are generally prescribed for four to eight weeks. However, it may be prescribed for patients with comorbidities and multiple medications for a longer period of time. While this remains true in terms of effectiveness, concerns have been raised about the safety of long-term PPI use and the serious adverse effects that may result. Some of the observational and population-based cohort studies have shown an association between long-term use of PPIs and an increased risk of pneumonia, major cardiovascular events, dementia, vitamin B12 deficiency, bone fractures, gastric cancer, and kidney injury, among others. This review analyzes the clinical data supporting the long-term use of PPIs and takes a deep dive into whether these several emerging long-term concerns apply to the currently available PPIs in India. We have summarized a vast array of studies, including randomized trials, cohort studies, and meta-analyses, that report low or high incidences of major health risks linked with PPIs and have assessed their appropriateness over a given period.

## Introduction and background

For more than 30 years, proton-pump inhibitors (PPIs) have revolutionized the management of acid peptic diseases due to their well-established efficacy and encouraging safety profile. Today, PPIs remain among the most widely prescribed medications for a variety of acid-related disorders, like gastroesophageal reflux disease (GERD), including oesophagitis and Barrett's esophagus, peptic ulcer disease and gastritis, and *Helicobacter pylori* (*H. pylori*) eradication (in conjunction with antibiotics). Additionally, they are frequently prescribed for prophylactic purposes and in conjunction with non-steroidal anti-inflammatory drugs (NSAIDs) [1,2]. PPIs are generally prescribed for four to eight weeks. However, healthcare providers also prescribe PPIs for patients with comorbidities and multiple medications for a longer period of time [3]. While this remains true in terms of effectiveness, there has been a growing concern over the potential safety issues associated with the long-term use of PPIs [4].

**Figure 1:**
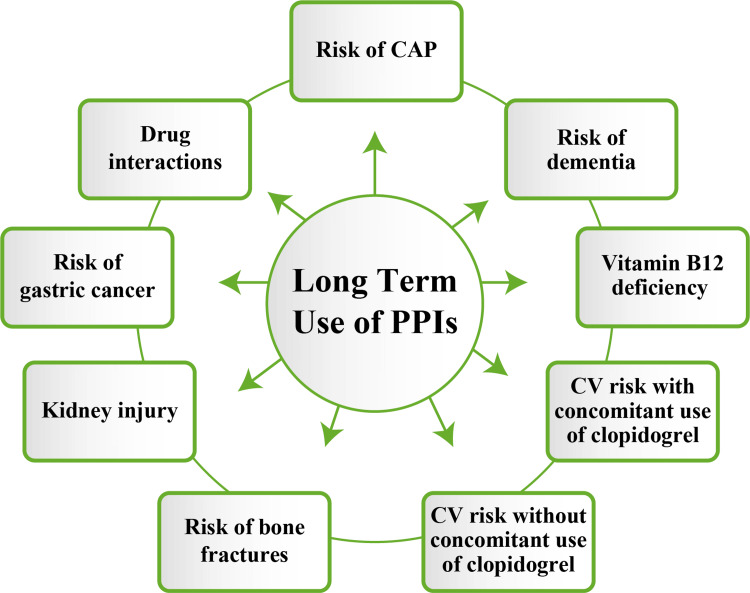
Potential safety concerns associated with long-term use of PPIs PPIs: proton-pump inhibitors; CV: cardiovascular; CAP: community-acquired pneumonia Source: Reference [4]

Several long-term observational and population-based cohort studies have shown an association between long-term use of PPIs and an increased risk of pneumonia, major cardiovascular (CV) events, dementia, vitamin B12 deficiency, bone fractures, gastric cancer, and kidney injury, among others (Figure 1) [4].

Subsequently, the Food and Drug Administration (FDA) has issued several safety warnings and raised concerns associated with long-term PPI use [5]. Notably, all currently available PPIs exhibit a range of both beneficial and potential adverse effects associated with their long-term use [6]. In this review, we sought to analyze the clinical data supporting the long-term use of PPIs and to evaluate whether these several emerging long-term concerns are applicable to the currently available PPIs (omeprazole, esomeprazole, pantoprazole, and rabeprazole).

## Review

Risk of community-acquired pneumonia with long-term use of PPIs

Although the causative mechanism of PPI-associated pneumonia is multifactorial, it is postulated to be caused by gastric colonization of acid-labile pathogenic bacteria, which is then aspirated due to a compromised stomach’s “acid mantle.” Another assumption is increased bacterial colonization of the larynx, esophagus, and lungs due to the inhibition of extra-gastric hydrogen potassium adenosine triphosphatase (H+/K+-ATPase) enzymes by PPIs, which may decrease the acidity of the upper aerodigestive tract [7]. Numerous trials have observed an association between PPI use and the development of community-acquired pneumonia (CAP). A meta-analysis of eight observational studies reported that the overall risk of pneumonia was increased by 27% with the use of PPIs [8]. However, a population-based case-control cohort study conducted for a five-year period in patients with gastrointestinal symptoms and an initial diagnosis of chronic kidney disease (CKD) showed that patients on long-term use of pantoprazole (12.1%) reported a lower risk of developing CAP than patients on rabeprazole (36.3%), omeprazole (25.8%), and esomeprazole (22.6%) [9].

Risk of dementia with long-term use of PPIs

Long-term use of PPIs has been hypothesized to hinder the activity of scavenger enzymes such as V-ATPase, leading to a buildup of beta-amyloid and thus causing the risk of progression and pathogenesis of dementia in humans [4]. Haenisch et al. examined a cohort of 3,076 patients aged 75 or older without prior dementia history. After accounting for confounding variables, they found that individuals using PPIs had a 38% higher risk of developing dementia and a 44% higher risk of Alzheimer's disease [10]. Gomm et al. conducted a comparable prospective cohort study involving 73,679 individuals aged 75 or older with no dementia at the study's outset. Their results likewise revealed a noteworthy 44% increased risk of dementia among those who regularly took PPI medications [11]. Three studies demonstrated a positive association between dementia and PPIs like omeprazole, esomeprazole, and lansoprazole, with an approximately 1.4-fold increased risk of any dementia in cohorts using PPIs (95% CI, 1.36-1.52; p<0.001) [12]. On the other hand, the largest trial with pantoprazole (>17,000 patients from 33 countries randomized to pantoprazole versus placebo, followed for a median of approximately three years) conducted by Moayyedi et al. showed no significant difference between pantoprazole and placebo for dementia (0.6% vs. 0.5%; p=0.36) [6].

Vitamin B12 deficiency with long-term use of PPIs

Long-term use of PPIs inhibits the production of gastric acid, which consequently decreases the digestive capacity to release vitamin B12 from foods and thus lowers the amount of vitamin B12 that is absorbed in the body [13]. A large case-control study compared 25,956 patients with vitamin B12 deficiency and 184,199 patients without vitamin B12 deficiency to assess the association with acid suppression therapy. Those who had received PPI treatment for more than two years reported a 65% increased risk of vitamin B12 deficiency when compared to those who did not [14]. However, only a slight decrease in vitamin B12 serum concentrations has been reported in a small number of patients receiving long-term therapy (>3 years) of omeprazole, but without any significant clinical concern [15]. On the other hand, based on an Indian study, vitamin B12 deficiency is highly prevalent in Indians, as up to 47% of subjects in the Indian population were reported to be vitamin B12 deficient [16]. Moreover, a retrospective data analysis showed no vitamin B12 deficiency in patients receiving concomitant metformin and PPI therapy of pantoprazole for one to four years [17].

CV risk with long-term use of PPIs and concomitant clopidogrel use

PPIs are frequently used in patients with CVs as prophylactic measures to mitigate the risk of gastrointestinal bleeding caused by antithrombotic or antiplatelet medications [14]. The latest guidelines of the European Society of Cardiology, the American College of Cardiology, and the American Heart Association have recommended PPIs to reduce the risk of bleeding complications in CV patients treated with dual antiplatelet therapy (DAPT) [18,19]. One of the early concerns regarding the use of PPIs was a potential interaction with the antiplatelet agent clopidogrel, a prodrug requiring activation by the cytochrome P450 isoenzyme 2C19 (CYP2C19). There has been concern that PPIs may decrease clopidogrel’s anti-platelet effect due to the competitive interaction between clopidogrel and PPIs with CYP2C19, affecting the clopidogrel-specific inhibition of ADP-induced platelet aggregation [20]. However, compelling evidence suggests that omeprazole has the highest affinity for CYP2C19, while pantoprazole has the lowest or no affinity [20]. In 2009, the US FDA suggested the avoidance of concomitant administration of clopidogrel and omeprazole due to a concern about a reduction in clopidogrel’s antiplatelet activity [4]. Likewise, esomeprazole is also primarily bioactivated by the CYP2C19 enzyme [20]. As a result, esomeprazole interferes with the clopidogrel pathway and may alter clopidogrel levels. Retrospective analyses of randomized trials of clopidogrel in patients with acute coronary syndromes revealed less benefit from the antiplatelet agent when esomeprazole was co-administered [21]. On the other hand, pantoprazole is metabolized by the CYP2C9 enzyme, which does not interfere with the CYP2C19 enzyme's function. As a result, clopidogrel levels remain unchanged. Furthermore, a Choi et al. study published in 2017 demonstrated that pantoprazole can be safely used as a PPI in patients receiving DAPT of aspirin and clopidogrel, as it does not reduce the antiplatelet effect of clopidogrel [22].

CV risk with long-term PPIs and without concomitant clopidogrel use

The influence of PPIs on CV risk in the absence of clopidogrel is not well established [23]. In a meta-analysis of nine randomized controlled trials (RCTs), Dahal et al. highlighted that regardless of clopidogrel use, PPI use alone does not increase the risks of all-cause mortality (p=0.31), CV mortality (p=0.30), nonfatal myocardial infarction/ischemia (p=0.22), ischemic stroke/transient ischemic attack (p=0.89) in patients taking long-term aspirin for prevention of cardiovascular diseases (CVDs), and stroke [20,24]. Likewise, a systemic review and meta-analysis (16 studies involving 447,408 participants) by Batchelor et al. also concluded there was no clear evidence of an association between PPI monotherapy and increased CV risk [23]. Jeridi et al., in their meta-analysis study, also highlighted no significant differences between the PPI group and the control group for the risks of major adverse CV events, all-cause death, and target vessel revascularization. Thus, this meta-analysis study also supported the hypothesis that there is no significantly increased risk of CV events in association with PPI use alone. In essence, these clinical studies suggest that initiation of PPI monotherapy should not be avoided because of CV risk [20].

The recent 76-week-extended phase, double-blind, and comparative study (PLANETARIUM study) with rabeprazole (10 or 5 mg once daily) also showed no clinically significant CV events in patients with a history of peptic ulcers [25]. Likewise, Moayyedi et al. conducted the largest trial with pantoprazole (>17,000 patients from 33 countries randomized to pantoprazole versus placebo, followed for a median of approximately three years) in patients with CVD/peripheral artery disease receiving rivaroxaban or aspirin. These study findings revealed no significant difference was observed in the primary efficacy outcome of the rivaroxaban/aspirin trial for the composite outcome of myocardial infarction, stroke, or CV deaths (hazard ratio (HR), 1.04; 95% CI, 0.93-1.15) with pantoprazole as compared to placebo. Moreover, hospitalization rates (HR, 1.04; 95% CI, 0.99-1.09) and all-cause mortality (HR, 1.03; 95% CI, 0.92-1.15) were also similar in the pantoprazole and placebo arms. Notably, this large placebo-controlled randomized trial concluded that long-term use of pantoprazole can be safely administered in patients with stable CVD [6].

Risk of bone fractures with long-term use of PPIs

The prolonged use of PPIs has been associated with a reduction in the enteral absorption of calcium [4]. Omeprazole, specifically, has been evaluated with tracer methods to demonstrate a reduction in the absorption of calcium carbonate in older women [4]. In a recent meta-analysis comprising 18 observational studies, it was found that the use of PPIs was linked to a modest increase in risk for fracture at any site (RR, 1.33; 95% CI, 1.15-1.54) and for risk of spine fracture (RR, 1.58; 95% CI, 1.38-1.82) [26].

A prospective cohort study by van der Hoorn et al. concluded that rabeprazole and esomeprazole were associated with increased fracture risk and osteoporosis in older women, and thus a proper benefit/risk assessment should be made when prescribing them [27]. On the other hand, Moayyedi et al. showed no significant difference between pantoprazole and placebo for fracture rate (2.3% vs. 2.4%; p=0.71) after a median follow-up of three years [6].

Kidney injury with long-term use of PPIs

The pathophysiological association between PPI use and CKD and whether PPI is the sole mechanism to increase the risk of acute interstitial nephritis-induced acute kidney disease remains unknown [4]. The results of the Atherosclerosis Risk in Communities study showed that individuals using PPIs had a 50% greater risk of CKD compared to those who did not use PPIs (n=10,482: with normal baseline renal function; median duration of 13.9 years). Nonusers followed a median duration of 13.9 years [28]. According to a study conducted by Al-Aly et al., PPI exposure was associated with an increased risk of developing CKD, progressing to CKD, and developing end-stage renal disease [3]. However, Thurber et al. demonstrated that an association between PPIs and adverse kidney outcomes is mostly derived from claims databases and/or observational studies and is not considered to be of a high level of quality. This study concluded that higher-quality data is required for a better understanding of long-term PPI-associated risks. Moreover, though PPIs should be used with vigilance, there is currently no need for wide adoption of de-escalation strategies with regard to long-term PPI use merely out of safety concerns [29]. The use of PPI cannot be the sole reason for renal damage when used concomitantly with nephrotoxic drugs [30]. Similarly, Moayyedi et al. also showed that chronic pantoprazole use had no significant between-group difference between pantoprazole and placebo for incident events of CKD (2.1% vs. 1.8%; p=0.15) [6].

Risk of gastric cancer with long-term use of PPIs

There are clinical concerns that significant acid inhibition with chronic PPI use may result in hypergastrinemia, resulting in endocrine cell hyperplasia and possible pre-neoplastic changes [31]. Compelling evidence suggests that the standardized incidence ratio of gastric cancer among PPI users is 3.38 (95% CI 3.23-3.53), and the risk of cancer increases with the duration of PPI use [32]. In a long-term follow-up investigation as mandated by the US FDA, involving 61,864 users of PPIs, the risk of gastric cancer was comparable between the use of pantoprazole and any combination of omeprazole, esomeprazole, lansoprazole, or rabeprazole (HR 0.68; 95% CI 0.24-1.93) after adjusting for factors such as age, gender, cumulative PPI dosage, H. pylori treatment, and the total duration of PPI therapy [33]. However, Brunner et al. revealed that long-term treatment with pantoprazole (for up to 15 years) is not associated with an increased risk of gastric malignancy. In this study, the histological evaluations of both the gastric corpus and antrum revealed no clinically relevant unfavorable changes over the course of 15 years of continuous treatment with pantoprazole [31].

Drug interactions with long-term use of PPIs

Another major concern is the interaction of PPIs with other drugs known to occur due to cytochrome P450 systems. Several studies showed that omeprazole carries considerable potential for drug interactions because of its high affinity for CYP2C19 and moderate affinity for CYP3A4 [34]. Dorofeeva et al. reported that omeprazole-treated patients with hypertension and acid-related disorders also on long-term amlodipine therapy may lead to a significantly stronger antihypertensive action [35]. On the other hand, rabeprazole has a weaker potential for interactions than omeprazole, though its interaction profile as compared to esomeprazole has been less extensively investigated [34]. Notably, pantoprazole is associated with lower incidences of drug interactions than older PPIs (omeprazole), resulting in lower affinity for specific CYP isoenzymes [34,36]. This is an important consideration, especially among patients with comorbidities who are affected by polypharmacy, which increases their risk of drug-drug interactions [36]. Moreover, most of the elderly have concomitant illnesses and receive other drugs, but this does not adversely affect the efficacy of pantoprazole because of its pharmacokinetics, which are independent of the patient’s age [15]. In essence, pantoprazole may be a preferred long-term treatment choice with fewer drug-drug interactions in the elderly or patients with comorbidities with fewer drug-drug interactions as compared to other PPIs [36].

## Conclusions

Considered safe and efficient drugs, since their introduction into clinical practice in the late 1990s, PPIs have shown significant positive effects on the treatment outcomes of patients with acid peptic disease. Though clinical evidence has highlighted multiple potential risks associated with long-term PPI use, the evidence is inconclusive and contradictory. Moreover, not all currently available PPIs, like pantoprazole, have been clinically proven to be associated with long-term potential risks. Nevertheless, long-term PPI should be used judiciously and with high clinical vigilance.
